# ﻿Five new troglobitic species of *Tyrannochthonius* (Arachnida, Pseudoscorpiones, Chthoniidae) from the Yunnan, Guizhou and Sichuan Provinces, China

**DOI:** 10.3897/zookeys.1131.91235

**Published:** 2022-11-23

**Authors:** Yun-Chun Li

**Affiliations:** 1 College of Life Science, China West Normal University, Nanchong, Sichuan 637009, China China West Normal University Nanchong China

**Keywords:** Cave-inhabiting, identification key, pseudoscorpion, soil-dwelling, taxonomy

## Abstract

Five new species of the genus *Tyrannochthonius* Chamberlin, 1929 are described from caves in the provinces of Yunnan (*T.huilongshanensis***sp. nov.**, *T.xinzhaiensis***sp. nov.**, and *T.yamuhensis***sp. nov.**), Guizhou (*T.dongjiensis***sp. nov.**), and Sichuan (*T.huaerensis***sp. nov.**). An identification key is provided for all known representatives of the genus *Tyrannochthonius* from China.

## ﻿Introduction

The pseudoscorpion tribe Tyrannochthoniini Chamberlin, 1962 belongs to the subfamily Chthoniinae Daday, 1889 and the family Chthoniidae Daday, 1889. It is distributed on all continents except Antarctica and contains six genera: *Lagynochthonius* Beier, 1951; *Maorichthonius* Chamberlin, 1925; *Paraliochthonius* Beier, 1956; *Troglochthonius* Beier, 1939; *Tyrannochthonius* Chamberlin, 1929; and *Vulcanochthonius* Muchmore, 2001 (World Pseudoscorpiones Catalog 2022). The tribe Tyrannochthoniini is characterized by one or two rows of chemosensory setae extending along the dorsum of the chelal hand; coxal spines are present only on coxae II; interior basal and interior sub-basal trichobothria situated slightly proximal of the middle of chelal hand; male sternite III elongated medially, with a very long notch (Judson 2007). Two of the genera, *Lagynochthonius* Beier, 1951 and *Tyrannochthonius* Chamberlin, 1929, have been reported in China.

The pseudoscorpion genus *Tyrannochthonius* was erected by Chamberlin for the Thai type species *Chthoniusterribilis* With, 1906 (by original designation) (Chamberlin 1929). The genus *Tyrannochthonius* is characterized by tergites V–IX each with eight setae at most; long coxal spines; apodeme of movable finger normal, not complex or strongly sclerotized; the sub-basal trichobothrium is positioned midway between sub-terminal and basal, or nearer to sub-terminal; chelal fingers usually straight in dorsal view; the hand of chela normal, not narrowed at base of fingers; chelal hand usually with a single large, medial acuminate spine-like seta near the base of the fixed finger, but this can be reduced or absent (Muchmore and Chamberlin 1995; Edward and Harvey 2008). During the identification of pseudoscorpion specimens collected from the Yunnan–Guizhou Plateau from 2017 to 2019, five new cave-inhabiting species of *Tyrannochthonius* were found, which are described in this article.

## ﻿Materials and methods

The specimens were preserved in 75% ethanol. They were cleared in lactic acid for 12–24 h at room temperature and, after the study, washed in distilled water and returned to alcohol. The specimens were examined with a Leica M205FA stereomicroscope and an Olympus CX31 compound microscope. Photographs were taken using a Canon 6D Mark II camera fitted with Laowa 25 mm f/2.8 2.5–5X and 100 mm F2.8 2.0X Ultra Macro lenses. The final high depth-of-field (DoF) images were stacked from 30 to 80 single photos using Helicon Focus 7.6.1., and CorelDRAW 2018 and SAI 2 softwares were used to draw the figures. The type specimens of the new species are deposited in the collection of the Museum of China West Normal University (**MCWNU**; Sichuan, China).

Pseudoscorpion terminology and measurements mostly follow Chamberlin (1931), with some minor modifications to the terminology of the trichobothria (Harvey 1992) and chelicera (Judson 2007).

## ﻿Systematic account

**Family Chthoniidae Daday, 1889**

**Subfamily Chthoniinae Daday, 1889**

**Tribe Tyrannochthoniini Chamberlin, 1962**

**Genus *Tyrannochthonius* Chamberlin, 1929**

### ﻿ Tyrannochthonius dongjiensis sp. nov.

Taxon classificationAnimaliaPseudoscorpionesChthoniidae

CE52411F-F7CD-5528-983C-7DCCCF7D887E

https://zoobank.org/B395357D-20CA-4B95-8423-9BC09EF468B4

Figs 1 , 6A, B

#### Type material.

***Holotype*** male: China, Guizhou Province, Luodian County, Dongjia Town, Dongjia Village, Nameless Cave, 25°38.53'N, 106°54.67'E, 869 m a.s.l., 7 October 2019, Yun-Chun Li leg., in MCWNU (Ar-Ps-GZ-0055). ***Paratypes***: 2 males, 4 females, collected with the holotype, in MCWNU (Ar-Ps-GZ-0008); 5 males, 2 females, Guizhou Province, Pingtang County, Tangbian Town, Baima Cave, 25°40'6.13"N, 106°45'53.89"E, 870 m a.s.l., 6 October 2019, Yun-Chun Li leg., in MCWNU (Ar-Ps-GZ-0010).

#### Diagnosis.

Troglobiont habitus. This new species is distinguished from other members of the genus *Tyrannochthonius* by the following combination of characters: carapace without eyes or eyespots, anterior margin with six setae; epistome absent; rallum composed of six blades; tergites I–IV with two setae; apex of coxa I with long and rounded anteromedial process, near the apex with a seta; chelal hand dorsal surface with chemosensory setae; fixed chelal finger with 24 or 25 teeth, movable chelal finger with 27–29 retrorse teeth. Pedipalpal femur (♂) 7.58–7.63×, (♀) 7.36–7.42× longer than broad, length (♂) 0.91–0.95 mm, (♀) 1.03–1.07 mm; chela (♂) 7.88–7.90×, (♀) 7.06–7.10 longer than deep, length (♂) 1.25–1.28 mm, (♀) 1.20–1.24 mm; ratio movable chelal finger/chelal hand (♂) 1.86–1.90×, (♀) 1.88–1.93×.

#### Etymology.

Latinized adjective, derived from the village of Dongjia, located near the type locality.

#### Description.

**Adult male** (Fig. 6A).

Pale yellow-orange, chelicera slightly darker, soft parts pale (Fig. 6A).

**Figure 1.**
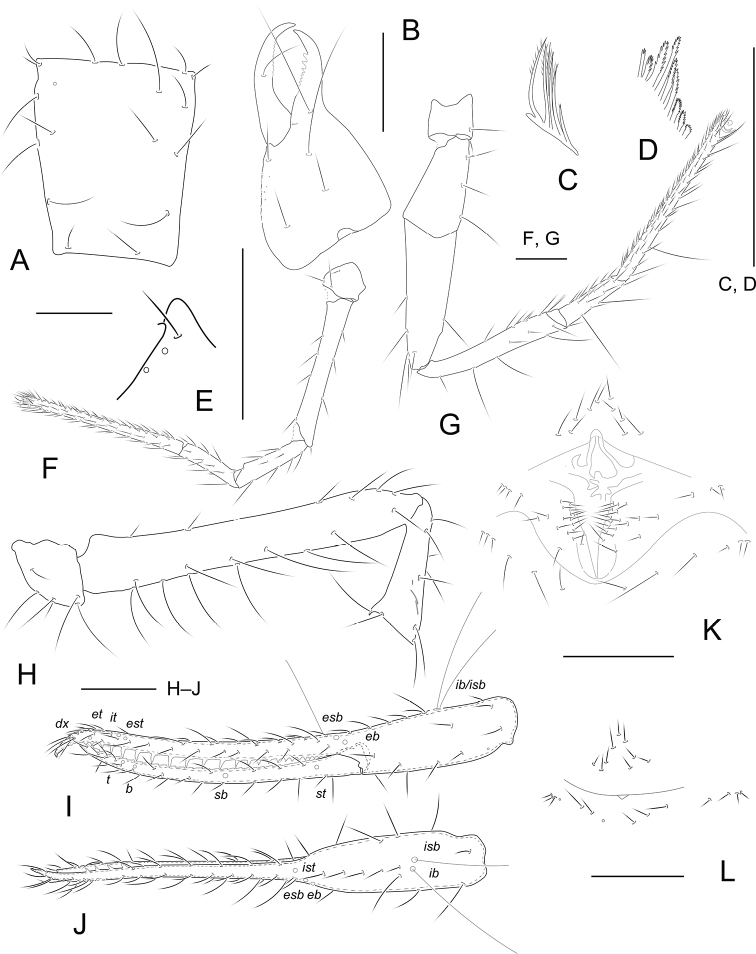
*Tyrannochthoniusdongjiensis* sp. nov., holotype male (**A–K**) and paratype female (**L**) **A** carapace **B** left chelicera **C** rallum of left chelicera **D** coxal spines **E** process of left coxa I, ventral view **F** left leg I, lateral view **G** left leg IV, lateral view **H** palp (minus chela) **I** chela, retrolateral view **J** chela, dorsal view **K** male genital area **L** female genital area. Scale bars: 0.20 mm

***Carapace*** (Fig. 1A): 1.26–1.30× longer than broad, no eyes or eyespots; epistome absent; carapace surface smooth, lateral margins distinctly constricted posteriorly. With 18 setae arranged 6: 4: 4: 2: 2, anterolateral setae much shorter than others. ***Coxae***: manducatory process pointed, with two distal setae, one long and the other slightly shorter. Pedipalpal coxa with three setae, coxa I 3, II 4, III 5, IV 5; intercoxal tubercle absent. Apex of coxa I with long and rounded anteromedial process, near the apex with a seta (Fig. 1E); coxae II with nine terminally indented coxal spines on each side, set as an oblique row, longer spines present in the middle of the row, becoming shorter distally and proximally and incised for ~ ½ their length (Fig. 1D). ***Chelicera*** (Fig. 1B): 1.82–1.85× longer than broad, hand with five setae and one lyrifissure dorsally, movable finger with one submedial seta. Cheliceral hand with moderate hispid granulation dorsally. Fixed finger with eight or nine teeth, distal one largest, decreasing in size proximally; movable finger with 12 or 13 teeth; galea absent. Serrula exterior with 22–25 blades. Rallum composed of six blades (Fig. 1C), distal blade weakly recumbent basally, with fine barbules and set apart from the other blades, the latter tightly grouped and with long pinnae. ***Pedipalp*** (Fig. 1H–J): all setae acuminate. Trochanter 1.56–1.61×, femur 7.58–7.63×, patella 2.73–2.76× longer than broad and with three lyrifissures (Fig. 1H). Femur 2.22–2.31× longer than patella. Chela 7.88–7.90×, hand 2.63–2.66× longer than deep; movable chelal finger 1.86–1.90× longer than hand. Chelal hand dorsal surface with a single row of five chemosensory setae between *esb* and *ib*/*isb* trichobothria; distal paraxial seta of hand not enlarged. Fingers straight in dorsal view (Fig. 1J). Fixed finger with 24 or 25 teeth, middle ones larger than those at both ends; movable finger with 27–29 retrorse teeth (Fig. 1I). Venom apparatus absent. Fixed chelal finger with eight trichobothria and movable finger with four, *ib* and *isb* situated close together, submedially on dorsum of chelal hand; *eb*, *esb*, and *ist* forming a straight oblique row at base of fixed chelal finger; *it* slightly distal to *est*, situated subdistally; *et* slightly nearer to tip of fixed finger; *dx* situated distal to *et*; *sb* half-way between *st* and *b*; *b* and *t* situated subdistally, *t* situated at same level as *est.****Opisthosoma***: tergites and sternites undivided; setae uniseriate and acuminate. Tergal chaetotaxy (I–XII): 2: 2: 2: 2: 3–4: 4: 4: 4: 4: 2: 0; sternal chaetotaxy (IV–XII): 12: 7: 7: 7: 7: 7: 7: 0: 2. Anterior genital operculum with ten setae, genital opening slit-like, with 14 or 15 setae on the right side and 18 on the left (Fig. 1K). ***Legs*** (Fig. 1F, G): leg I: trochanter 1.00–1.03×, femur 7.63–7.66× longer than deep and 1.91–1.97× longer than patella; patella 4.57–4.59×, tibia 4.33–4.37×, tarsus 13.40–13.44× longer than deep. Leg IV: trochanter 0.88–0.96×, femoropatella 3.83–3.87×, tibia 6.89–6.92× longer than deep, basitarsus 3.75–3.80×longer than deep, with a basal tactile seta (TS = 0.24–0.25), telotarsus 15.2–15.5×longer than deep and 2.53–2.55× longer than basitarsus, with a tactile seta near base (TS = 0.23–0.24). Arolia on legs I and IV shorter than claws.

**Adult female** (Fig. 6B).

Mostly the same as the holotype with the differences listed below.

***Carapace***: slightly longer than broad (1.13–1.15×). ***Chelicera***: 2.30–2.33× longer than broad. ***Pedipalp***: trochanter 1.80–1.86× longer than broad, femur 7.36–7.42× longer than broad, patella 2.63–2.70× longer than broad, femur 2.45–2.49× longer than patella. Chela 7.06–7.10× longer than deep, hand 2.41–2.46× longer than deep; movable finger 1.88–1.93× longer than hand. ***Opisthosoma***: tergal chaetotaxy (I–XII): 2: 2: 2: 2: 4: 4: 4: 4: 4: 2: 2: 0; sternal chaetotaxy (IV–XII): 14: 12: 8: 7: 7: 9: 7: 0: 2. Anterior genital operculum with 9 + 14 setae on posterior margin (Fig. 1L).

***Dimensions*** (mm, length/width or, in the case of the legs, chela, and chelal hand, length/depth).

Males (females in parentheses): body length 2.24–2.30 (2.49–2.56). Carapace 0.54–0.57/0.43–0.45 (0.53–0.55/0.47–0.48). Pedipalp: trochanter 0.25–0.28/0.16–0.18 (0.27–0.29/0.15–0.17), femur 0.91–0.95/0.12–0.14 (1.03–1.07/0.14–0.16), patella 0.41–0.44/0.15–0.17 (0.42–0.44/0.16–0.18), hand 0.42–0.45/0.16–0.17 (0.41–0.44/0.17–0.18), length of movable chelal finger 0.78–0.80 (0.77–0.79), chela 1.25–1.28/0.16–0.17 (1.20–1.24/0.17–0.18). Chelicera: 0.51–0.53/0.28–0.29 (0.53–0.55/0.23–0.24). Leg I: trochanter 0.15–0.17/0.15–0.16 (0.15–0.17/0.14–0.16), femur 0.61–0.64/0.08–0.09 (0.62–0.65/0.08–0.09), patella 0.32–0.35/0.07–0.08 (0.31–0.34/0.07–0.08), tibia 0.26–0.27/0.06–0.07 (0.27–0.29/0.06–0.07), tarsus 0.67–0.69/0.05–0.06 (0.66–0.68/0.05–0.06). Leg IV: trochanter 0.15–0.17/0.17–0.18 (0.17–0.19/0.14–0.16), femoropatella 0.88–0.92/0.23–0.25 (0.90–0.93/0.22–0.24), tibia 0.62–0.65/0.09–0.10 (0.63–0.65/0.09–0.10), basitarsus 0.30–0.32/0.08–0.09 (0.29–0.31/0.08–0.09), telotarsus 0.76–0.79/0.05–0.06 (0.79–0.82/0.06–0.07).

#### Distribution.

China (Guizhou).

### ﻿ Tyrannochthonius huaerensis sp. nov.

Taxon classificationAnimaliaPseudoscorpionesChthoniidae

3A3D2D92-6B2C-517A-9C88-5B1629E048F5

https://zoobank.org/E7DE51E4-4C4D-41E5-A949-9095DB01128E

Figs 2 , 6C, D

#### Type material.

***Holotype*** male: China, Sichuan Province, Luzhou City, Gulin County, Shipping Town, Xiangding Village, Huaer Cave, 28°02.22'N, 106°01.43'E, 760 m a.s.l., 3 November 2019, Yun-Chun Li leg., in MCWNU (Ar-Ps-SC-0052). ***Paratypes***: 4 males, 2 females, collected with the holotype, in MCWNU (Ar-Ps-SC-0001).

#### Diagnosis.

Troglobiont habitus. This new species is distinguished from other members of the genus *Tyrannochthonius* by the following combination of characters: carapace without eyes or eyespots, anterior margin with four setae; epistome very small; rallum composed of eight blades; tergites I–VI with four setae; chelal finger without intercalary teeth; coxae II with 12 terminally indented coxal spines on each side; chelal hand dorsal surface with chemosensory setae; apex of coxa I with long and rounded anteromedial process, near the apex without setae; movable finger retrolateral margins weakly curved between *st* and *sb* trichobothria; fixed chelal finger with 23 or 24 teeth, movable chelal finger with 14 or 15 macrodenticles and 7–9 vestigial teeth. Pedipalpal femur (♂) 8.92–8.95×, (♀) 8.54–8.59× longer than broad, length (♂) 1.16–1.19 mm, (♀) 1.11–1.17 mm; chela (♂) 7.00–7.07×, (♀) 8.67–8.69× longer than deep, length (♂) 1.61–1.64 mm, (♀) 1.56–1.58 mm; ratio movable chelal finger/chelal hand (♂) 1.56–1.59×, (♀) 1.52–1.55×.

#### Etymology.

Latinized adjective, derived from the type locality, namely Huaer Cave.

#### Description.

**Adult male** (Fig. 6C).

Carapace, chelicera, pedipalps, and tergites I–VI reddish brown, remaining parts yellowish brown (Fig. 6C).

**Figure 2.**
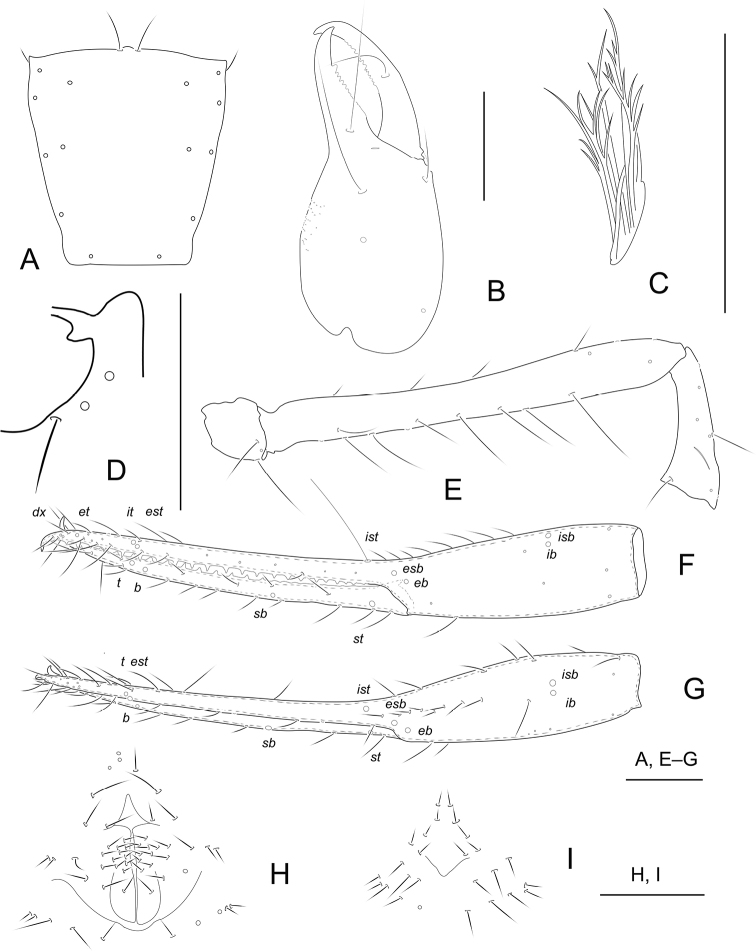
*Tyrannochthoniushuaerensis* sp. nov., holotype male (**A–H**) and paratype female (**I**) **A** carapace **B** right chelicera **C** rallum of left chelicera **D** process of left coxa I, ventral view **E** palp (minus chela) **F** chela, retrolateral view **G** chela, dorsal view **H** male genital area **I** female genital area. Scale bars: 0.20 mm.

***Carapace*** (Fig. 2A): 1.11–1.13× longer than broad, no eyes or eyespots; epistome very small, triangular; carapace surface smooth, lateral margins distinctly constricted posteriorly. With 18 setae arranged 4: 6: 4: 2: 2, anterolateral setae much shorter than others. ***Coxae***: manducatory process pointed, with two distal setae, one long and the other slightly shorter. Pedipalpal coxa with three setae, coxa I 3, II 4, III 5, IV 5; intercoxal tubercle absent. Apex of coxa I with long and rounded anteromedial process, near the apex without setae (Fig. 2D); coxae II with 12 terminally indented coxal spines on each side, set as an oblique row, longer spines present in the middle of the row, becoming shorter distally and proximally and incised for ~ ½ their length. ***Chelicera*** (Fig. 2B): 2.31–2.33× longer than broad, hand with five setae and one lyrifissure dorsally, movable finger with one submedial seta. Cheliceral hand with moderate hispid granulation dorsally. Fixed finger with 12 or 13 teeth, distal one largest, decreasing in size proximally; movable finger with 13 or 14 teeth; galea absent. Serrula exterior with 20–22 blades. Rallum composed of eight blades (Fig. 2C), distal blade weakly recumbent basally, with fine barbules and set apart from the other blades, the latter tightly grouped and with long pinnae. ***Pedipalp*** (Fig. 2E–G): all setae acuminate. Trochanter 1.25–1.30×, femur 8.92–8.95×, patella 2.75–2.78× longer than broad and with one lyrifissure. Femur 2.64–2.70× longer than patella. Chela 7.00–7.07×, hand 2.74–2.76× longer than deep; movable chelal finger 1.56–1.59× longer than hand. Chelal hand dorsal surface with a single row of seven chemosensory setae between *esb* and *ib*/*isb* trichobothria; distal paraxial seta of hand not enlarged. Fingers straight in dorsal view (Fig. 2G). Fixed finger with 23 or 24 teeth, middle ones larger than those at both ends; movable finger with 14 or 15 macrodenticles, base of finger with 7–9 very low, vestigial teeth (Fig. 2F). Venom apparatus absent. Movable finger retrolateral margins weakly curved between *st* and *sb* trichobothria. Fixed chelal finger with eight trichobothria and movable finger with four, *ib* and *isb* situated close together, submedially on dorsum of chelal hand; *eb*, *esb*, and *ist* forming a straight oblique row at base of fixed chelal finger; *it* slightly distal to *est*, situated subdistally; *et* slightly nearer to tip of fixed finger; *dx* situated distal to *et*; *sb* near to *st*; *b* and *t* situated subdistally, *t* situated at same level as *it.****Opisthosoma***: tergites and sternites undivided; setae uniseriate and acuminate. Tergal chaetotaxy (I–XII): 4: 4: 4: 4: 4: 4: 3: 3: 4: 4: 2: 0; sternal chaetotaxy (IV–XII): 10: 10: 9: 9: 9: 11: 8: 0: 2. Anterior genital operculum with ten setae, genital opening slit-like, with 14 or 15 marginal setae on each side (Fig. 2H). ***Legs***: leg I: trochanter 1.58–1.59×, femur 8.25–8.30× longer than deep and 2.00–2.04× longer than patella; patella 4.71–4.75×, tibia 4.14–4.18×, tarsus 11.17–11.20× longer than deep. Leg IV: trochanter 1.15–1.18×, femoropatella 3.33–3.39×, tibia 6.70–6.72× longer than deep, basitarsus 3.75–3.79× longer than deep, with a basal tactile seta (TS = 0.21–0.22), telotarsus 12.67–12.70× longer than deep and 2.53–2.55× longer than basitarsus, with a tactile seta near base (TS = 0.19–0.20). Arolia on legs I and IV shorter than claws.

**Adult female** (Fig. 6D).

Mostly the same as the holotype with the differences listed below.

***Carapace***: slightly longer than broad (1.08–1.10×). ***Chelicera***: 2.27–2.29× longer than broad. ***Pedipalp***: trochanter 1.81–1.84× longer than broad, femur 8.54–8.59× longer than broad, patella 2.87–2.89× longer than broad, femur 2.58–2.60× longer than patella. Chela 8.67–8.69× longer than deep, hand 3.44–3.47× longer than deep; movable finger 1.52–1.55× longer than hand. ***Opisthosoma***: tergal chaetotaxy (I–XII): 4: 4: 4: 4: 4: 4: 4: 4: 5: 4: 2: 0; sternal chaetotaxy (IV–XII): 10: 9: 9: 8: 10: 10: 8: 0: 2. Anterior genital operculum with 10 + 6 setae on posterior margin (Fig. 2I).

***Dimensions*** (mm, length/width or, in the case of the legs, chela, and chelal hand, length/depth).

Males (females in parentheses): body length 1.89–1.95 (1.87–1.99). Carapace 0.59–0.60/0.53–0.54 (0.53–0.55/0.49–0.51). Pedipalp: trochanter 0.20–0.22/0.16–0.18 (0.29–0.30/0.16–0.17), femur 1.16–1.19/0.13–0.15 (1.11–1.17/0.13–0.15), patella 0.44–0.45/0.16–0.17 (0.43–0.44/0.15–0.16), hand 0.63–0.65/0.23–0.25 (0.62–0.65/0.18–0.20), length of movable chelal finger 0.98–0.99 (0.94–0.97), chela 1.61–1.64/0.23–0.25 (1.56–1.58/0.18–0.20). Chelicera: 0.60–0.61/0.26–0.28 (0.59–0.60/0.26–0.27). Leg I: trochanter 0.19–0.20/0.12–0.14 (0.19–0.21/0.12–0.14), femur 0.66–0.69/0.08–0.09 (0.57–0.59/0.08–0.09), patella 0.33–0.35/0.07–0.08 (0.30–0.32/0.07–0.08), tibia 0.29–0.30/0.07–0.08 (0.29–0.30/0.06–0.07), tarsus 0.67–0.69/0.06–0.07 (0.65–0.67/0.05–0.06). Leg IV: trochanter 0.23–0.25/0.20–0.21 (0.17–0.19/0.14–0.16), femoropatella 0.90–0.92/0.27–0.29 (0.83–0.86/0.25–0.27), tibia 0.67–0.69/0.10–0.11 (0.62–0.64/0.10–0.11), basitarsus 0.30–0.32/0.08–0.09 (0.27–0.29/0.08–0.09), telotarsus 0.76–0.79/0.06–0.07 (0.70–0.74/0.05–0.06).

#### Distribution.

China (Sichuan).

### ﻿ Tyrannochthonius huilongshanensis sp. nov.

Taxon classificationAnimaliaPseudoscorpionesChthoniidae

AC143CFC-F7F1-590C-920E-16FBBA7E3D27

https://zoobank.org/E1FC5D11-2ACA-4ED1-9750-A71BA3248737

Figs 3 , 7A, B

#### Type material.

***Holotype*** male: China, Yunnan Province, Dali City, Nanjian County, Xiaowan Town, Huilongshan Village, Banpoyan Cave, 24°56.01'N, 100°18.87'E, 1990 m a.s.l., 23 August 2018, Yun-Chun Li leg., in MCWNU (Ar-Ps-YN-0079). ***Paratypes***: 2 males, 7 females, collected with the holotype, in MCWNU (Ar-Ps-YN-0012).

#### Diagnosis.

Troglobiont habitus. This new species is distinguished from other members of the genus *Tyrannochthonius* by the following combination of characters: carapace without eyes or eyespots, anterior margin with four- setae; epistome present; tergites I–V with four setae; coxae II with eight terminally indented coxal spines on each side; apex of coxa I with long and rounded anteromedial process, near the apex with a seta; chelal hand dorsal surface with chemosensory setae; fixed chelal finger with 28 teeth and 16 or 17 intercalary teeth, movable chelal finger with 15 or 16 macrodenticles, 12 or 13 intercalary teeth and 5–7 vestigial teeth. Pedipalpal femur (♂) 4.87–4.90×, (♀) 5.33–5.37× longer than broad, length (♂) 0.73–0.76 mm, (♀) 0.80–0.83 mm; chela (♂) 5.61–5.66×, (♀) 6.37–6.40× longer than deep, length (♂) 1.01–1.09 mm, (♀) 1.21–1.25 mm; ratio movable chelal finger/chelal hand (♂) 1.75–1.80×, (♀) 1.80–1.83×.

#### Etymology.

Latinized adjective, derived from the village of Huilongshan, which is near the type locality.

#### Description.

**Adult male** (Fig. 7A).

Chelicera reddish brown, remaining parts yellowish brown (Fig. 7A).

**Figure 3.**
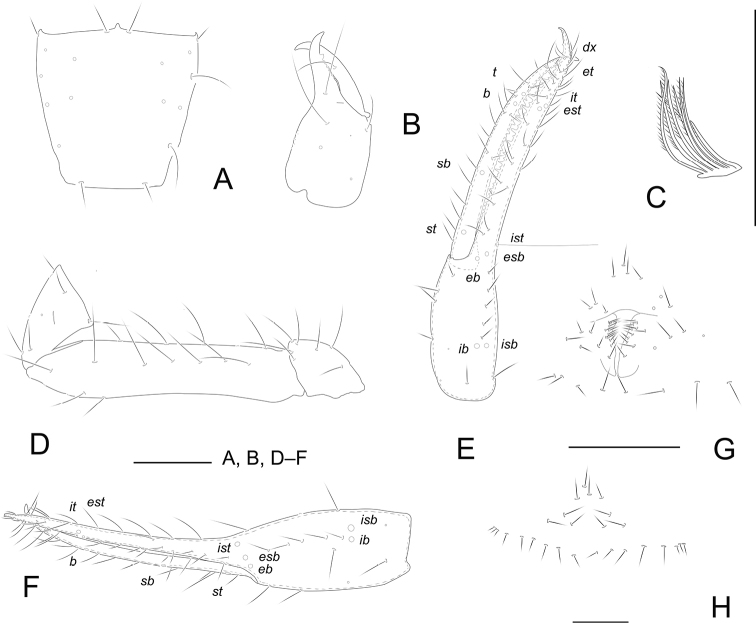
*Tyrannochthoniushuilongshanensis* sp. nov., holotype male (**A–G**) and paratype female (**H**) **A** carapace **B** right chelicera **C** rallum of left chelicera **D** palp (minus chela) **E** chela, retrolateral view **F** chela, dorsal view **G** male genital area **H** female genital area. Scale bars: 0.20 mm.

***Carapace*** (Fig. 3A): 1.02–1.06× longer than broad, no eyes or eyespots; epistome small, triangular, with two setae flanking base; carapace surface smooth, lateral margins distinctly constricted posteriorly. With 18 setae arranged 4: 6: 4: 2: 2, anterolateral setae much shorter than others. ***Coxae***: manducatory process pointed, with two distal setae, one long and the other slightly shorter. Pedipalpal coxa with three setae, coxa I 3, II 4, III 5, IV 5; intercoxal tubercle absent. Apex of coxa I with long and rounded anteromedial process, near the apex with a seta; coxae II with eight terminally indented coxal spines on each side, set as an oblique row, longer spines present in the middle of the row, becoming shorter distally and proximally and incised for ~ ½ their length. ***Chelicera*** (Fig. 3B): 2.25–2.29× longer than broad, hand with five setae and one lyrifissure dorsally, movable finger with one submedial seta. Cheliceral hand with moderate hispid granulation dorsally. Fixed finger with eight or nine teeth, distal one largest, decreasing in size proximally; movable finger with 7–9 teeth; galea absent. Serrula exterior with 19–21 blades. Rallum composed of eight blades (Fig. 3C), distal blade weakly recumbent basally, with fine barbules and set apart from the other blades, the latter tightly grouped and with long pinnae. ***Pedipalp*** (Fig. 3D–F): all setae acuminate. Trochanter 1.60–1.61×, femur 4.87–4.90×, patella 1.61–1.64× longer than broad and with one lyrifissure. Femur 2.52–2.55× longer than patella. Chela 5.61–5.66×, hand 2.00–2.10× longer than deep; movable chelal finger 1.75–1.80× longer than hand. Chelal hand dorsal surface with a single row of five chemosensory setae between *esb* and *ib*/*isb* trichobothria; distal paraxial seta of hand not enlarged. Fingers straight in dorsal view (Fig. 3F). Fixed finger with 28 teeth and 16 or 17 intercalary teeth, middle ones larger than those at both ends; movable finger with 15 or 16 macrodenticles and 12 or 13 intercalary teeth, base of finger with 5–7 very low, vestigial teeth (Fig. 3E). Venom apparatus absent. Fixed chelal finger with eight trichobothria and movable finger with four, *ib* and *isb* situated close together, submedially on dorsum of chelal hand; *eb*, *esb*, and *ist* forming a straight oblique row at base of fixed chelal finger; *it* slightly distal to *est*, situated subdistally; *et* slightly nearer to tip of fixed finger; *dx* situated distal to *et*; *sb* near to *st*; *b* and *t* situated subdistally, *t* situated at same level as *it.****Opisthosoma***: tergites and sternites undivided; setae uniseriate and acuminate. Tergal chaetotaxy (I–XII): 4: 4: 4: 4: 4: 6: 6: 6: 6: 4: 2: 0; sternal chaetotaxy (IV–XII): 8: 10: 6: 6: 6: 7: 7: 0: 2. Anterior genital operculum with ten setae, genital opening slit-like, with 11 or 12 marginal setae on each side (Fig. 3G). ***Legs***: leg I: trochanter 1.68–1.70×, femur 6.50–6.58× longer than deep and 1.56–1.59× longer than patella; patella 4.17–4.20×, tibia 4.00–4.06×, tarsus 8.40–8.47× longer than deep. Leg IV: trochanter 1.00–1.07×, femoropatella 2.59–2.63×, tibia 4.40–4.47× longer than deep, basitarsus 2.71–2.74× longer than deep, with a basal tactile seta (TS = 0.15–0.17), telotarsus 9.60–9.66× longer than deep and 2.53–2.55× longer than basitarsus, with a tactile seta near base (TS = 0.15–0.16). Arolia on legs I and IV shorter than claws.

**Adult female** (Fig. 7B).

Mostly the same as the holotype with the differences listed below.

***Carapace***: slightly longer than broad (0.90–0.99×). ***Chelicera***: 2.13–2.17× longer than broad. ***Pedipalp***: trochanter 1.86–1.88× longer than broad, femur 5.33–5.37× longer than broad, patella 1.94–1.98× longer than broad, femur 2.58–2.59× longer than patella. Chela 6.37–6.40× longer than deep, hand 2.11–2.15× longer than deep; movable finger 1.80–1.83× longer than hand. ***Opisthosoma***: tergal chaetotaxy (I–XII): 4: 4: 4: 4: 5: 6: 6: 6: 5: 4: 2: 0; sternal chaetotaxy (IV–XII): 12: 10: 7: 8: 7: 7: 6: 0: 2. Anterior genital operculum with 10 + 17 setae on posterior margin (Fig. 3H).

***Dimensions*** (mm, length/width or, in the case of the legs, chela, and chelal hand, length/depth).

Males (females in parentheses): body length 1.68–1.75 (1.89–1.95). Carapace 0.44–0.46/0.43–0.44 (0.45–0.49/0.50–0.51). Pedipalp: trochanter 0.24–0.26/0.15–0.17 (0.26–0.28/0.14–0.16), femur 0.73–0.76/0.15–0.17 (0.80–0.83/0.15–0.17), patella 0.29–0.31/0.18–0.19 (0.31–0.33/0.16–0.17), hand 0.36–0.40/0.18–0.20 (0.40–0.44/0.19–0.20), length of movable chelal finger 0.63–0.67 (0.72–0.76), chela 1.01–1.09/0.18–0.20 (1.21–1.25/0.19–0.20). Chelicera: 0.45–0.47/0.20–0.22 (0.51–0.54/0.24–0.26). Leg I: trochanter 0.17–0.19/0.10–0.11 (0.16–0.18/0.14–0.15), femur 0.39–0.42/0.06–0.07 (0.45–0.46/0.08–0.09), patella 0.25–0.27/0.06–0.07 (0.28–0.30/0.07–0.08), tibia 0.20–0.22/0.05–0.06 (0.22–0.25/0.06–0.07), tarsus 0.42–0.45/0.05–0.06 (0.49–0.53/0.05–0.06). Leg IV: trochanter 0.16–0.17/0.16–0.17 (0.21–0.22/0.15–0.17), femoropatella 0.57–0.59/0.22–0.24 (0.54–0.57/0.20–0.22), tibia 0.44–0.46/0.10–0.11 (0.43–0.46/0.11–0.12), basitarsus 0.19–0.21/0.07–0.08 (0.21–0.23/0.08–0.09), telotarsus 0.48–0.50/0.05–0.06 (0.50–0.54/0.05–0.06).

#### Distribution.

China (Sichuan).

### ﻿ Tyrannochthonius xinzhaiensis sp. nov.

Taxon classificationAnimaliaPseudoscorpionesChthoniidae

57632123-F87B-584F-9B82-A154580AB684

https://zoobank.org/3DFBBE98-7B37-4AB6-851E-6256660A4F9F

Figs 4 , 7C, D

#### Type material.

***Holotype*** male: China, Yunnan Province, Zhaotong City, Zhenxiong County, Wude Town, Xinzhai Village, Daguoquan Cave, 27°35.90'N, 104°46.25'E, 1301 m a.s.l., 8 April 2017, Yun-Chun Li leg., in MCWNU (Ar-Ps-YN-0080). ***Paratypes***: 1 male, 6 females, 6 tritonymphs, collected with the holotype, in MCWNU (Ar-Ps-YN-0007).

#### Diagnosis.

Troglobiont habitus. This new species is distinguished from other members of the genus *Tyrannochthonius* by the following combination of characters: carapace without eyes or eyespots, anterior margin with five or six setae; epistome present; tergites V–X with four setae; coxae II with 12 terminally indented coxal spines on each side; apex of coxa I with long and rounded anteromedial process, near the apex without setae; chelal hand dorsal surface with chemosensory setae; fixed chelal finger with 26 teeth, movable chelal finger with 34 or 35 teeth. Pedipalpal femur (♂) 6.94–6.97×, (♀) 6.71–6.77× longer than broad, length (♂) 1.18–1.21 mm, (♀) 1.14–1.18 mm; chela (♂) 7.90–7.91×, (♀) 6.44–6.42× longer than deep, length (♂) 1.66–1.68 mm, (♀) 1.61–1.64 mm; ratio movable chelal finger/chelal hand (♂) 1.61–1.64×, (♀) 1.76–1.80×.

#### Etymology.

Latinized adjective, derived from the village of Xinzhai, located near the type locality.

#### Description.

**Adult male** (Fig. 7C).

Carapace and chelicera reddish brown, remaining parts yellowish brown (Fig. 7C).

**Figure 4.**
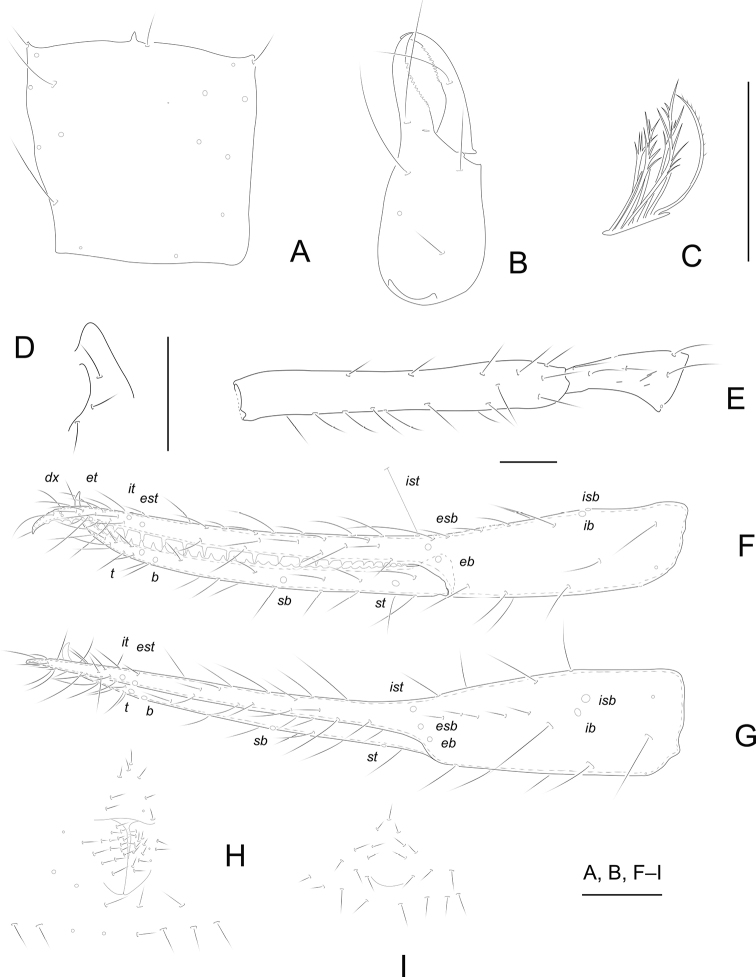
*Tyrannochthoniusxinzhaiensis* sp. nov., holotype male (**A–H**) and paratype female (**I**) **A** carapace **B** right chelicera **C** rallum of left chelicera **D** process of left coxa I, ventral view **E** palp (minus chela) **F** chela, retrolateral view **G** chela, dorsal view **H** male genital area **I** female genital area. Scale bars: 0.20 mm.

***Carapace*** (Fig. 4A): 0.98–1.01× longer than broad, no eyes or eyespots; epistome very pointed and small, triangular; carapace surface smooth, lateral margins weakly constricted posteriorly. With 17 or 18 setae arranged 5–6: 4: 4: 2: 2, anterolateral setae much shorter than others. ***Coxae***: manducatory process pointed, with two distal setae, one long and the other slightly shorter. Pedipalpal coxa with three setae, coxa I 3, II 4, III 5, IV 5; intercoxal tubercle absent. Apex of coxa I with long and rounded anteromedial process, near the apex without setae; coxae II with 12 terminally indented coxal spines on each side, set as an oblique row, longer spines present in the middle of the row, becoming shorter distally and proximally and incised for ~ ½ their length. ***Chelicera*** (Fig. 4B): 2.59–2.61× longer than broad, hand with five setae and one lyrifissure dorsally, movable finger with one submedial seta. Cheliceral hand with moderate hispid granulation dorsally. Fixed finger with 16 teeth, distal one largest, decreasing in size proximally; movable finger with 14 or 15 teeth; galea absent. Serrula exterior with 23 or 24 blades. Rallum composed of eight blades (Fig. 4C), distal blade weakly recumbent basally, with fine barbules and set apart from the other blades, the latter tightly grouped and with long pinnae. ***Pedipalp*** (Fig. 4E–G): all setae acuminate. Trochanter 1.56–1.59×, femur 6.94–6.97×, patella 1.83–1.86× longer than broad and with four lyrifissures. Femur 2.68–2.70× longer than patella. Chela 7.90–7.91×, hand 2.90–2.93× longer than deep; movable chelal finger 1.61–1.64× longer than hand. Chelal hand dorsal surface with a single row of five chemosensory setae between *esb* and *ib*/*isb* trichobothria; distal paraxial seta of hand not enlarged. Fingers straight in dorsal view (Fig. 4G). Fixed finger with 26 teeth, middle ones larger than those at both ends; movable finger with 34 or 35 teeth (Fig. 4F). Venom apparatus absent. Fixed chelal finger with eight trichobothria and movable finger with four, *ib* and *isb* situated close together, submedially on dorsum of chelal hand; *eb*, *esb*, and *ist* forming a straight oblique row at base of fixed chelal finger; *it* slightly distal to *est*, situated subdistally; *et* slightly nearer to tip of fixed finger; *dx* situated distal to *et*; *sb* near to *st*; *b* and *t* situated subdistally, *t* situated at same level as *est.****Opisthosoma***: tergites and sternites undivided; setae uniseriate and acuminate. Tergal chaetotaxy (I–XII): 4: 4: 4: 4: 4: 4: 4: 4: 4: 4: 2: 0; sternal chaetotaxy (IV–XII): 12: 10: 10: 9: 9: 9: 7: 0: 2. Anterior genital operculum with nine setae, genital opening slit-like, with 15 or 16 marginal setae on each side (Fig. 4H). ***Legs***: leg I: trochanter 1.43–1.44×, femur 6.60–6.62× longer than deep and 1.78–1.79× longer than patella; patella 4.11–4.14×, tibia 3.88–3.92×, tarsus 9.57–9.60× longer than deep. Leg IV: trochanter 1.05–1.07×, femoropatella 3.83–3.85×, tibia 5.91–5.93× longer than deep, basitarsus 3.75–3.76× longer than deep, with a basal tactile seta (TS = 0.20–0.21), telotarsus 12.17–12.20× longer than deep and 2.43–2.45× longer than basitarsus, with a tactile seta near base (TS = 0.18–0.19). Arolia on legs I and IV shorter than claws.

**Adult female** (Fig. 7D).

Mostly the same as the holotype with the differences listed below.

***Carapace***: slightly longer than broad (1.00–1.02×). With 18 setae, including six on the anterior margin and two on the posterior margin. ***Chelicera***: 2.26–2.27× longer than broad. ***Pedipalp***: trochanter 1.82–1.86× longer than broad, femur 6.71–6.77× longer than broad, patella 1.76–1.79× longer than broad, femur 3.08–3.12× longer than patella. Chela 6.44–6.42× longer than deep, hand 2.20–2.22× longer than deep; movable finger 1.76–1.80× longer than hand. ***Opisthosoma***: tergal chaetotaxy (I–XII): 4: 4: 3: 3: 4: 4: 4: 4: 4: 4: 2: 0; sternal chaetotaxy (IV–XII): 14: 10: 9: 9: 9: 9: 7: 0: 2. Anterior genital operculum with 9 + 6 setae on posterior margin (Fig. 4I).

***Dimensions*** (mm, length/width or, in the case of the legs, chela, and chelal hand, length/depth).

Males (females in parentheses): body length 2.76–2.85 (2.69–2.88). Carapace 0.59–0.61/0.60–0.61 (0.58–0.60/0.58–0.59). Pedipalp: trochanter 0.25–0.26/0.16–0.17 (0.31–0.34/0.17–0.19), femur 1.18–1.21/0.17–0.19 (1.14–1.18/0.17–0.19), patella 0.44–0.46/0.24–0.26 (0.37–0.40/0.21–0.23), hand 0.61–0.63/0.21–0.22 (0.55–0.58/0.25–0.26), length of movable chelal finger 0.98–1.00 (0.97–1.01), chela 1.66–1.68/0.21–0.22 (1.61–1.64/0.25–0.26). Chelicera: 0.70–0.73/0.27–0.29 (0.61–0.64/0.27–0.29). Leg I: trochanter 0.20–0.22/0.14–0.15 (0.16–0.18/0.14–0.15), femur 0.66–0.68/0.10–0.11 (0.62–0.65/0.08–0.09), patella 0.37–0.39/0.09–0.10 (0.31–0.34/0.06–0.07), tibia 0.31–0.32/0.08–0.09 (0.31–0.33/0.06–0.07), tarsus 0.67–0.69/0.07–0.08 (0.64–0.67/0.06–0.07). Leg IV: trochanter 0.21–0.23/0.20–0.21 (0.19–0.21/0.17–0.19), femoropatella 0.92–0.95/0.24–0.26 (0.83–0.86/0.22–0.24), tibia 0.65–0.67/0.11–0.12 (0.57–0.60/0.09–0.10), basitarsus 0.30–0.31/0.08–0.09 (0.27–0.29/0.07–0.08), telotarsus 0.73–0.75/0.06–0.07 (0.68–0.70/0.05–0.06).

#### Distribution.

China (Yunnan).

### ﻿ Tyrannochthonius yamuhensis sp. nov.

Taxon classificationAnimaliaPseudoscorpionesChthoniidae

3358A2FE-1778-5D47-9EE8-7F6B445D5E02

https://zoobank.org/0DBC3106-021A-4D81-BC44-92B6D00E600C

Figs 5 , 8A

#### Type material.

***Holotype*** male: China, Yunnan Province, Lushui City, Fugong County, Shiyueliang Town, Lishadi Village, Yamu River, Nameless Cave, 27°07.69'N, 98°51.61'E, 1500 m a.s.l., 18 August 2018, Yun-Chun Li leg., in MCWNU (Ar-Ps-YN-0078). ***Paratypes***: 1 male, collected with the holotype, in MCWNU (Ar-Ps-YN-0014).

#### Diagnosis

(male, female unknown). Troglobiont habitus. This new species is distinguished from other members of the genus *Tyrannochthonius* by the following combination of characters: carapace without eyes or eyespots, anterior margin with four setae; epistome present; tergites II–VI with four setae; coxae II with ten terminally indented coxal spines on each side; apex of coxa I with long and rounded anteromedial process, near the apex with a seta; chelal hand dorsal surface with chemosensory setae. Fixed chelal finger with 25 teeth and 20 intercalary teeth, movable chelal finger with 22–24 teeth and three or four intercalary teeth. Pedipalpal femur 6.06–6.07× longer than broad, length 0.97–0.99 mm; chela 7.63–7.66× longer than deep, length 1.45–1.46 mm; ratio movable chelal finger/chelal hand 1.91–1.92×.

#### Etymology.

Latinized adjective, derived from the river of Yamuhe, which is near the type locality.

#### Description.

**Adult male** (Fig. 8).

Chelicera reddish brown, carapace and opisthosoma brown, remaining parts yellowish brown (Fig. 8).

**Figure 5.**
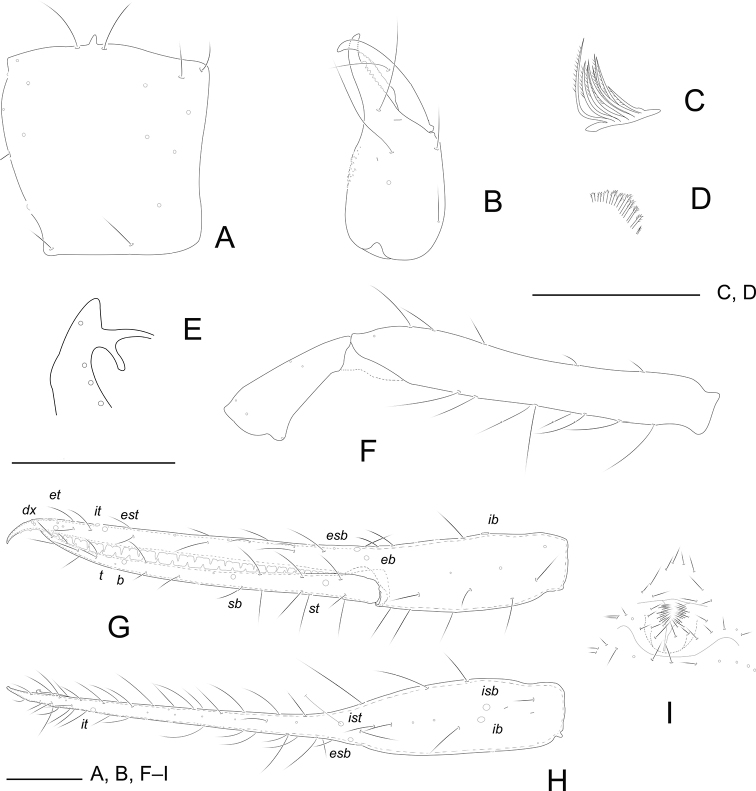
*Tyrannochthoniusyamuhensis* sp. nov., holotype male (**A–I**) **A** carapace **B** right chelicera **C** rallum of left chelicera **D** coxal spines **E** process of right coxa I, ventral view **F** palp (minus chela) **G** chela, retrolateral view **H** chela, dorsal view **I** male genital area. Scale bars: 0.20 mm.

***Carapace*** (Fig. 5A): 1.06–1.08× longer than broad, no eyes or eyespots; epistome very pointed and small, triangular; carapace surface smooth, lateral margins weakly constricted posteriorly. With 18 setae, including four on anterior margin and two on posterior margin, anterolateral setae much shorter than others. ***Coxae***: manducatory process pointed, with two distal setae, one long and the other slightly shorter. Pedipalpal coxa with three setae, coxa I 3, II 4, III 5, IV 5; intercoxal tubercle absent. Apex of coxa I with long and rounded anteromedial process, near the apex with a seta; coxae II with ten terminally indented coxal spines on each side, set as an oblique row, longer spines present in the middle of the row, becoming shorter distally and proximally and incised for ~ ½ their length (Fig. 5D). ***Chelicera*** (Fig. 5B): 2.31–2.33× longer than broad, hand with five setae and two lyrifissures dorsally, movable finger with one submedial seta. Cheliceral hand with moderate hispid granulation dorsally. Fixed finger with eight or nine teeth, distal one largest, decreasing in size proximally; movable finger with 12 or 13 teeth; galea absent. Serrula exterior with 20 or 21 blades. Rallum composed of eight blades (Fig. 5C), distal blade weakly recumbent basally, with fine barbules and set apart from the other blades, the latter tightly grouped and with long pinnae. ***Pedipalp*** (Fig. 5F–H): all setae acuminate. Trochanter 1.01–1.04×, femur 6.06–6.07×, patella 2.38–2.40× longer than broad. Femur 2.55–2.56× longer than patella. Chela 7.63–7.66×, hand 2.47–2.50× longer than deep; movable chelal finger 1.91–1.92× longer than hand. Chelal hand dorsal surface with a single row of five chemosensory setae between *esb* and *ib*/*isb* trichobothria; distal paraxial seta of hand not enlarged. Fingers straight in dorsal view (Fig. 5H). Fixed finger with 25 teeth and 20 intercalary teeth, middle ones larger than those at both ends; movable finger with 22–24 teeth and three or four intercalary teeth (Fig. 5G). Venom apparatus absent. Fixed chelal finger with eight trichobothria and movable finger with four, *ib* and *isb* situated close together, submedially on dorsum of chelal hand; *eb*, *esb*, and *ist* forming a straight oblique row at base of fixed chelal finger; *it* slightly distal to *est*, situated subdistally; *et* slightly nearer to tip of fixed finger; *dx* situated distal to *et*; *sb* near to *st*; *b* and *t* situated subdistally, *t* situated at same level as *est.****Opisthosoma***: tergites and sternites undivided; setae uniseriate and acuminate. Tergal chaetotaxy (I–XII): 3: 4: 4: 4: 4: 4: 6: 5: 5: 5: 2: 0; sternal chaetotaxy (IV–XII): 12: 10: 7: 7: 7: 7: 6: 0: 2. Anterior genital operculum with ten, genital opening slit-like, with 15 or 16 marginal setae on each side (Fig. 5I). ***Legs***: leg I: trochanter 1.38–1.40×, femur 6.63–6.65× longer than deep and 1.77–1.79× longer than patella; patella 4.29–4.30×, tibia 5.20–5.22×, tarsus 11.80–11.81× longer than deep. Leg IV: trochanter 1.06–1.07×, femoropatella 3.00–3.02×, tibia 5.70–5.71× longer than deep, basitarsus 3.00–3.01× longer than deep, with a basal tactile seta (TS = 0.20–0.21), telotarsus 12.80–12.81× longer than deep and 2.67–2.69× longer than basitarsus, with a tactile seta near base (TS = 0.19–0.20). Arolia on legs I and IV shorter than claws.

***Dimensions*** (mm, length/width or, in the case of the legs, chela, and chelal hand, length/depth).

Males: body length 2.25–2.30. Carapace 0.56–0.57/0.53–0.54. Pedipalp: trochanter 0.14–0.15/0.14–0.15, femur 0.97–0.99/0.16–0.18, patella 0.38–0.39/0.16–0.17, hand 0.47–0.49/0.19–0.20, length of movable chelal finger 0.90–0.92, chela 1.45–1.46/0.19–0.20. Leg I: trochanter 0.18–0.19/0.13–0.15, femur 0.53–0.55/0.08–0.09, patella 0.30–0.31/0.07–0.08, tibia 0.26–0.28/0.05–0.06, tarsus 0.59–0.60/0.05–0.06. Leg IV: trochanter 0.18–0.20/0.17–0.18, femoropatella 0.75–0.77/0.25–0.26, tibia 0.57–0.59/0.10–0.11, metatarsus 0.24–0.25/0.08–0.09, tarsus 0.64–0.66/0.05–0.06.

**Figure 6.**
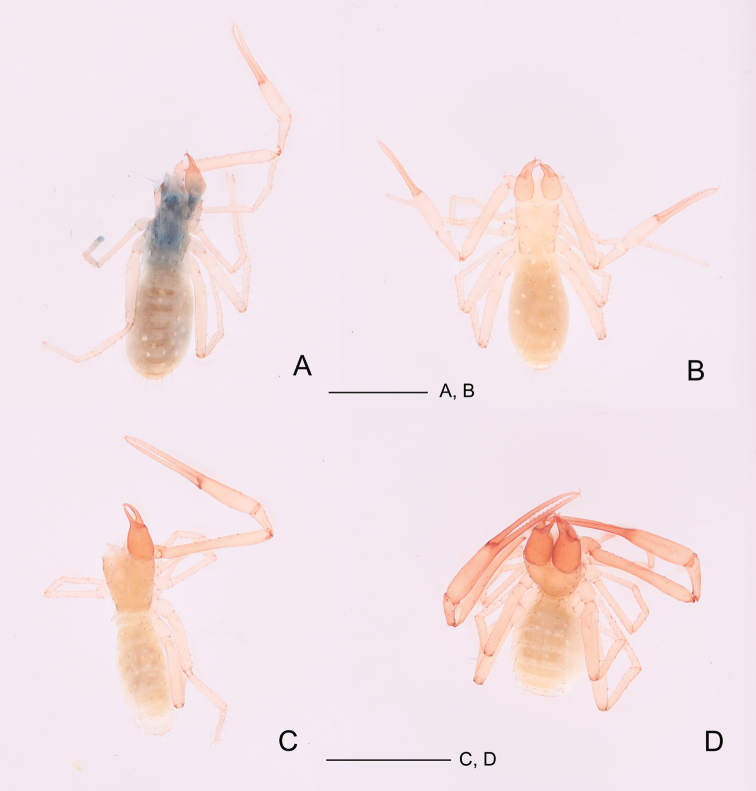
**A, B***Tyrannochthoniusdongjiensis* sp. nov., dorsal views **A** holotype male **B** paratype female **C, D***T.huaerensis* sp. nov., dorsal views **C** holotype male **D** paratype female. Scale bar: 1.00 mm (**A–D**).

**Figure 7.**
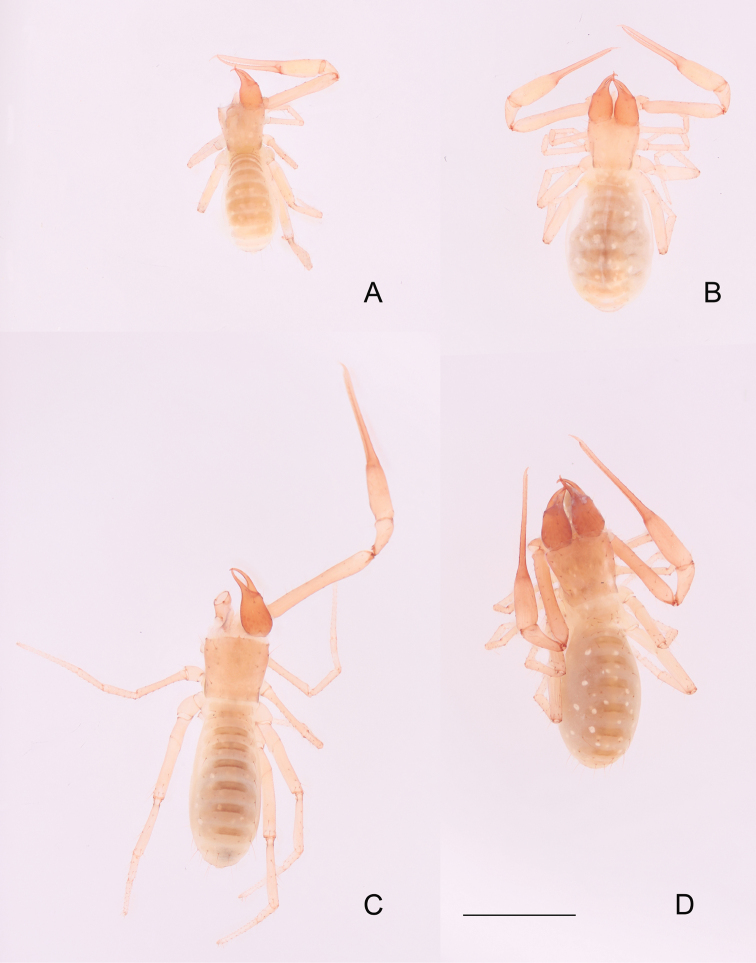
**A, B***Tyrannochthoniushuilongshanensis* sp. nov., dorsal views **A** holotype male **B** paratype female **C, D***T.xinzhaiensis* sp. nov., dorsal views **C** holotype male **D** paratype female. Scale bar: 1.00 mm (**A–D**).

#### Distribution.

China (Yunnan).

### ﻿Key to the species of *Tyrannochthonius* known from China (subspecies not included)

**Table:** 

1	Carapace with eyes	**2**
–	Carapace without eyes or eyespots	**4**
2	Chelal finger without intercalary teeth	**3**
–	Chelal finger with intercalary teeth	***T.robustus* Beier, 1951**
3	Carapace with 18 setae; tergites VIII–IX each with 8 setae	***T.japonicus* (Ellingsen, 1907)**
–	Carapace with 16 setae; tergites VIII–IX each with 6 setae	***T.pachythorax* Redikorzev, 1938**
4	Chelal finger with intercalary teeth	**5**
–	Chelal finger without intercalary teeth	**11**
5	Intercalary teeth only present on chelal finger	**6**
–	Intercalary teeth present on both chelal fingers	**7**
6	Rallum with 6 pinnate blades; coxae II with 5 or 6 terminally indented coxal spines on each side; epistome present	***T.zhai* Gao, Zhang & Chen, 2020**
–	Rallum with 7 or 8 pinnate blades; coxae II with 7 terminally indented coxal spines on each side; epistome absent	***T.chixingi* Gao, Wynne & Zhang, 2018**
7	Carapace anterior margin with 6 setae; chemosensory setae absent	**8**
–	Carapace anterior margin with 4 setae; chemosensory setae present	**10**
8	Tergites I–II each with 2 setae	**9**
–	Tergites I–II each with 4 setae	***T.antridraconis* Mahnert, 2009**
9	Palpal femur 6.60× as long as broad (length 0.90 mm), chela 7.70× longer than deep	***T.akaleus* Mahnert, 2009**
–	Palpal femur 5.90–6.70× as long as broad (length 0.95–0.97 mm), chela 6.90–7.30× longer than deep	***T.ganshuanensis* Mahnert, 2009**
10	Coxae II with 8 terminally indented coxal spines on each side ; chela 5.61–5.66× longer than deep	***huilongshanensis* sp. nov.**
–	Coxae II with 10 terminally indented coxal spines on each side; chela 7.63–7.66× longer than deep	***T.yamuhensis* sp. nov.**
11	Chelal fingers straight in dorsal view	**12**
–	Chelal fingers gently curved in dorsal view	***T.pandus* Hou, Gao & Zhang, 2022**
12	Chelal movable fingers without retrorse teeth; epistome present	**13**
–	Chelal movable fingers with retrorse teeth; epistome absent	***T.dongjiensis* sp. nov.**
13	Carapace anterior margin with 4 setae	**14**
–	Carapace anterior margin with 5 or 6 setae	***T.xinzhaiensis* sp. nov.**
14	Coxae II with 8 terminally indented coxal spines on each side; rallum with 6 pinnate blades	***T.harveyi* Gao, Zhang & Chen, 2020**
–	Coxae II with 12 terminally indented coxal spines on each side; rallum with 8 pinnate blades	***T.huaerensis* sp. nov.**

## ﻿Discussion

There are 146 known species of *Tyrannochthonius*, including four subspecies, of which 52 species live in caves. Other than China, these cave species are distributed in Africa, Oceania, and North America. Among them, there are 31 species in the United States, five species in Australia, four species in Mexico, one species in Kenya, one species in New Caledonia, one species in Guatemala, one species in Peru, and one species in Jamaica (Hou et al. 2022; World Pseudoscorpiones Catalog 2022).

In China, ten species and one subspecies have been recorded (Fig. 9), including seven cave-dwelling species, three species and one subspecies that are soil-dwelling: *T.akaleus* Mahnert, 2009 (Chuandongzi Cave) and *T.antridraconis* Mahnert, 2009 (Perte du Dragon Cave) from Chongqing; *T.ganshuanensis* Mahnert, 2009 (Changcao Cave) from Hubei; *T.chixingi* Gao, Wynne & Zhang, 2018 (Maomaotou Cave) from Guangxi; *T.dongjiensis* sp. nov. (Nameless Cave and Baima Cave), *T.harveyi* Gao, Zhang & Chen, 2020 (Yutang Cave) and *T.zhai* Gao, Zhang & Chen, 2020 (Jiangjia Cave) from Guizhou; *T.huaerensis* sp. nov. (Huaer Cave) from Sichuan; *T.huilongshanensis* sp. nov. (Banpoyan Cave), *T.pandus* Hou, Gao & Zhang, 2022 (Biyun Cave), *T.xinzhaiensis* sp. nov. (Daguoquan Cave) and *T.yamuhensis* sp. nov. (Nameless Cave) from Yunnan; *T.japonicus* (Ellingsen, 1907) and *T.japonicusjaponicus* (Ellingsen, 1907), soil-dwelling species from Yunnan and Taiwan; *T.pachythorax* Redikorzev, 1938, a soil-dwelling species from Yunnan, Sichuan, and Fujian; and *T.robustus* Beier, 1951 a soil-dwelling species from Sichuan, Zhejiang, Hunan, and Shaanxi (Schawaller 1995; Mahnert 2009; Gao et al. 2018, 2020; Hou et al. 2022). The eyes of these cave-dwelling species are completely degraded.

**Figure 8.**
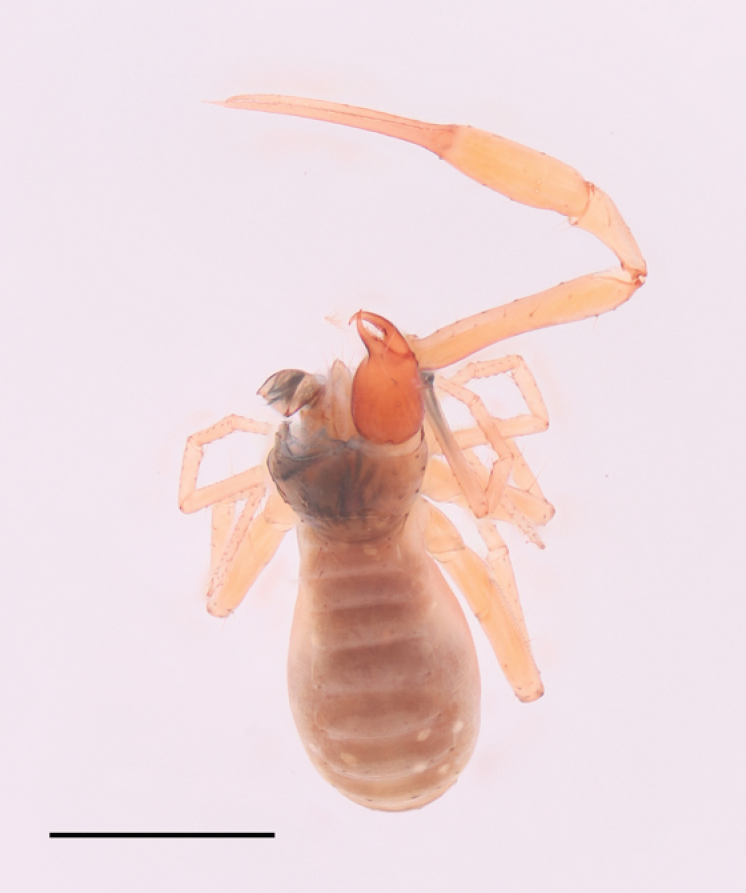
*Tyrannochthoniusyamuhensis* sp. nov., dorsal view, holotype male. Scale bar: 1.00 mm.

**Figure 9.**
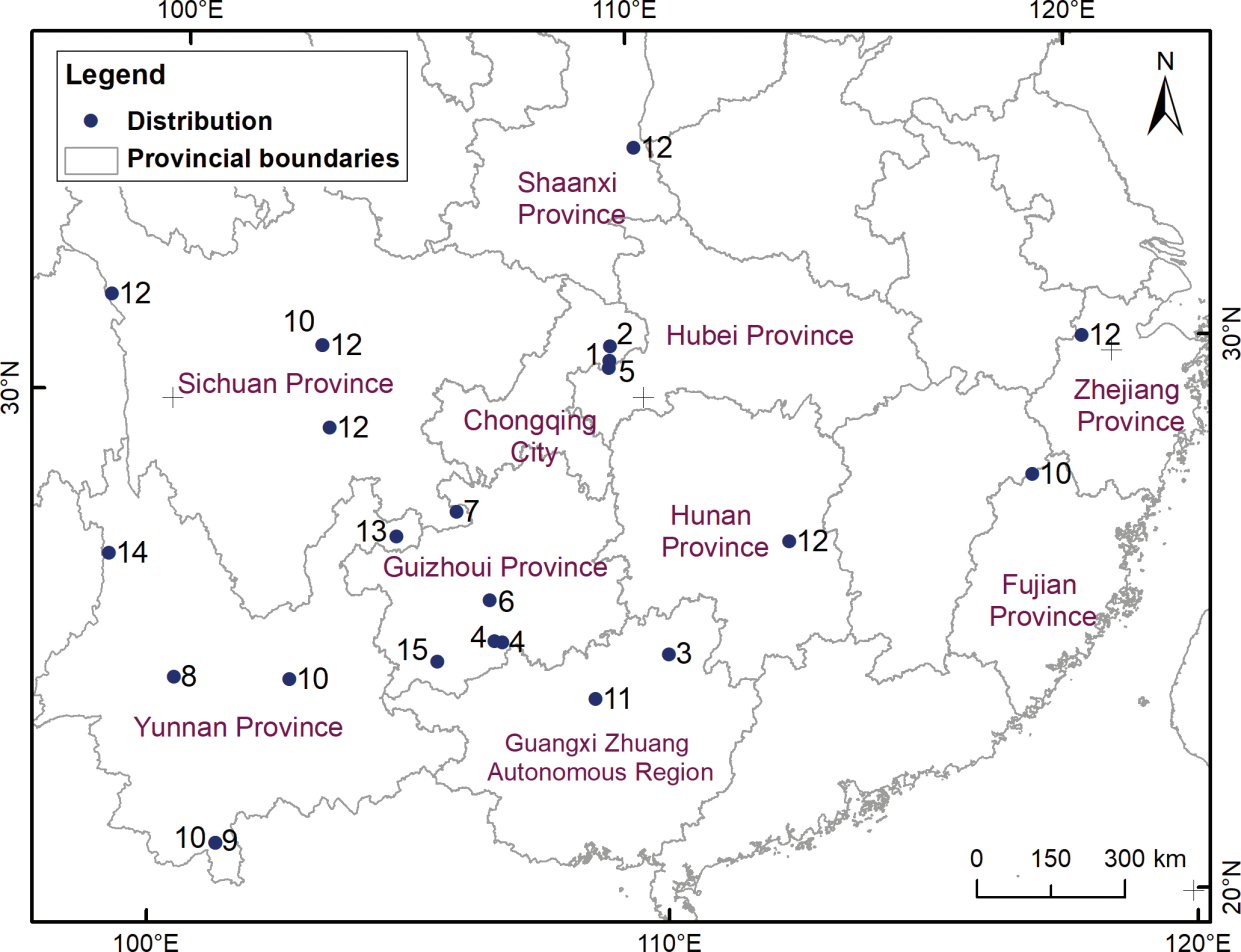
Known distribution of genus *Tyrannochthonius* from China. 1 *T.akaleus*; 2 *T.antridraconis*; 3 *T.chixingi*; 4 *T.dongjiensis* sp. nov.; 5 *T.ganshuanensis*; 6 *T.harveyi*; 7 *T.huaerensis* sp. nov.; 8 *T.huilongshanensis* sp. nov.; 9 *T.japonicus*; 10 *T.pachythorax*; 11 *T.pandus*; 12 *T.robustus*; 13 *T.xinzhaiensis* sp. nov.; 14 *T.yamuhensis* sp. nov.; 15 *T.zhai*.

The five new cave-dwelling species are easily distinguished from the seven known species: the chelal fingers of all new species are straight in dorsal view, while in *T.pandus* they are slightly curved. The movable finger of *T.dongjiensis* sp. nov. has retrorse teeth, which is similar to that of *T.zhai*, but the new species have a carapace with 18 setae and tergites I–IV each with two setae; the latter carapace only with 16 setae, and tergites I–IV each with four setae. There are only 16 setae on the carapace of *T.chixingi*, the other species have 17 or 18 setae. *T.huaerensis* sp. nov., *T.huilongshanensis* sp. nov., *T.xinzhaiensis* sp. nov., and *T.yamuhensis* sp. nov. are different from the remaining species (except *T.antridraconis*) in that the new species have tergites I-II each with three or four setae, while the latter only has two setae. In the new species, the chelal hand presents chemosensory setae on the dorsum, while in *T.antridraconis* they are absent. *T.huilongshanensis* sp. nov. and *T.yamuhensis* sp. nov. have intercalary teeth, the former with ten coxal spines and chela 7.63–7.66× longer than broad; in the latter, with eight coxal spines and chela 5.61–5.66× longer than broad. In *T.huaerensis* sp. nov., the anterior margin of the carapace with four setae, a slender and pointed epistome, palpal femur 8.92–8.95× as long as broad, and movable finger retrolateral margins weakly curved between *st* and *sb* trichobothria; in contrast, in *T.xinzhaiensis* sp. nov. the anterior margin of the carapace with five or six setae, epistome very small, palpal femur 6.94–6.97× as long as broad, movable finger retrolateral margins straight between *st* and *sb* trichobothria. In the known species, the chemosensory setae on the dorsal surface of the chelal hand are absent, while in the new species, there is a row of five to seven setae on the dorsal surface of the chelal hand.

## Supplementary Material

XML Treatment for Tyrannochthonius dongjiensis

XML Treatment for Tyrannochthonius huaerensis

XML Treatment for Tyrannochthonius huilongshanensis

XML Treatment for Tyrannochthonius xinzhaiensis

XML Treatment for Tyrannochthonius yamuhensis
